# MicroRNA-506 has a suppressive effect on the tumorigenesis of nonsmall-cell lung cancer by regulating tubby-like protein 3

**DOI:** 10.1080/21655979.2021.2001216

**Published:** 2021-12-07

**Authors:** Zhan-Hua Li, Ji-Hong Zhou, Si-Ning Chen, Ling Pan, Yuan Feng, Mei-Qun Luo, Rui-Xiang Li, Gui-Li Sun

**Affiliations:** a Department of Respiratory and Critical Care Medicine, Ruikang Hospital Affiliated to Guangxi Traditional Chinese Medicine University, Nanning, China; b Department of Respiratory Medicine, Shenzhen Baoan Hospital of Traditional Chinese Medicine, Shenzhen, China

**Keywords:** MicroRNA-506, nonsmall-cell lung cancer, tubby-like protein 3, mitochondrial apoptosis

## Abstract

MicroRNA-506 (miR-506), a miRNA, has been proven to act as a tumor suppressor gene in nonsmall-cell lung cancer (NSCLC); Tubby-like protein 3 (TULP3) is a potential target gene of miR-506. This study investigates whether miR-506 can prevent NSCLC progression by mediating TULP3. In vivo and in vitro experiments were performed to explore the function and potential regulatory relationship of miR-506 and TULP3 in NSCLC. Our results revealed that miR-506 is high expression in NSCLC cell lines, and the overexpression of miR-506 could inhibit cell viability and enhance cell apoptosis in H1299 and A549 cells. Pro-apoptotic related protein (cytochrome C, Bax, and cleaved caspase-9) expression increased while anti-apoptotic related protein (BCL-2 and BCL-XL) expression decreased after miR-506 was overexpression. Meanwhile, the overexpression of miR-506 could notably downregulate TULP3. Additionally, silence of TULP3 inhibited cell viability and promoted cell apoptosis. At the same time, pro-apoptotic related protein expression was promoted while anti-apoptotic related protein expression was inhibited. Furthermore, TULP3 overexpression could markedly reverse the inhibitory effect of miR-506 on the proliferation and induction of mitochondrial apoptosis in H1299 and A549 cells. In vivo tumor formation experiments also exhibited consistent results indicating that the functions of TULP3 might be correlated with the promotion of tumorigenesis. In conclusion, we firstly found that miR-506 can be involved in the processes of NSCLC and exert a suppressive effect on tumorigenesis by regulating TULP3 expression.

## 1. Introduction

Lung cancer is a common disease worldwide. As recorded, it causes more than 1.6 million deaths annually worldwide [1]. Approximately, 85% of the cases were attributed to nonsmall-cell lung cancer (NSCLC) [2]. However, the 5-year survival rate of NSCLC remains very low at ~15% [3]. Therefore, there is a need to develop effective oncological diagnostic solutions and treatment for NSCLC.

MicroRNAs (miRNAs) are a type of RNA that regulates gene expression [4,5]. Mature miRNA can bind to the target mRNA at the complementary sites at the 3ʹ-UTR or coding regions to inhibit the expression of the target genes [5]. Currently, it has been reported that miRNAs are involved in human cancer and carcinogenesis processes [6]. Since specific miRNAs are found in the differential expressions of specific cancers, miRNA expression profiles can assist in cancer classification, diagnosis, and clinical prognostic evaluation [7]. Regarding NSCLC, it has been reported that some miRNAs exhibited deregulation during the pathogenic processes [8]. The miR-34 family was shown to be involved in the p53 processes and suppress tumorigenesis in diverse cancers, including NSCLC [9,10]. Downregulated expressed let-7a and upregulated expressed miR-155 are associated with poor clinical outcomes in NSCLC [11]. It has been reported that miR-506 can inhibit the cell cycle progression and angiogenesis of NSCLC cells [12]. Therefore, miR-506 can play a crucial role in regulating the processes correlated with NSCLC. However, the specific mechanism of miR-506 in NSCLC progression remains unclear.

Through bioinformatics analysis, it was unexpectedly found that tubby-like protein 3 (TULP3) may be the target gene of miR-506. TULP3 is one of the protein families with tubby domains at the C-terminal, which plays a crucial role as a transcription factor [13]. Moreover, TULP3 plays a vital role in embryo development, which is associated with neural tube defects and embryo death [14]. Besides, TULP3 has been reported to modulate the carcinogenesis of pancreatic ductal adenocarcinoma (PDAC) [15]. Further, TULP3 has been suggested as a marker for colorectal cancer [16]. Moreover, research has proved that TULP3 can affect the processes of abdominal aortic aneurysm, including apoptosis and proliferation [17]. However, the function and possible mechanism of TULP3 in NSCLC have not been clearly elucidated. It is also unclear if miR-506 can affect the proliferation and apoptosis of NSCLC cells by targeting TULP3.

Therefore, we speculated that miR-506 maybe inhibite NSCLC progress by regulating TULP3. To verify our hypothesis, the influence of miR-506 on the viability and mitochondrial apoptosis of NSCLC cells was further confirmed. Moreover, the expression of TULP3 in NSCLC and the relationship between miR-506 and TULP3 were analyzed. The role of TULP3 in the viability and mitochondrial apoptosis of NSCLC cells was also explored. Furthermore, the rescue experiment was performed to confirm the function of miR-506 and TULP3 in vivo and in vitro. Our study is the first time to demonstrate that miR-506 inhibited the development of NSCLC by regulating TULP3. Therefore, our study provided a new clues for NSCLC progression.

## 2. Materials and methods

### 2.1. Cell culture

The MRC-5, A549, NCI-H1299 (H1299), HCC827, NCI-H23, PC9, NCI-H385, and 293 T cell lines were bought from the American Type Culture Collection (Manassas, VA, USA). They were cultured in a humidified 5% CO_2_ in Dulbecco’s Modified Eagle Medium supplemented with 10% fetal bovine serum.

### 2.2. Cell transfection

As indicated in previous studies [18], negative control of miR-506 mimics (NC), miR-506 mimics, siRNA negative control (siRNA-NC), and siRNA-TULP3 were designed and obtained from GenePharma (Shanghai, China). The core sequences of TULP3 were synthesized and cloned into the vector pcDNA3.1 (+). Empty vector pcDNA3.1 (+) was used as the control. The transfection for the plasmid, mimics, and siRNAs was conducted using lipofectamine 3000 according to the manufacturer’s instructions (Thermo Fisher, Waltham, MA, USA).

### 2.3. MTS assay

As manifested in previous studies [19], the cells (1 × 10^5^) in 100-μl medium were seeded into 96-well plates. At the indicated time, the 3-(4,5-dimethylthiazol-2-yl)-5-(3-carboxymethoxyphenyl)-2-(4- sulfophenyl)-2 H-tetrazolium (MTS) (Promega, Madison, WI, USA) was added and cultured for 3 h; the optical density of the cells at 490 nm was then detected in a microplate reader (Molecular Devices, Sunnyvale, CA, USA).

### 2.4. Cell apoptosis assay

As shown in previous literature [20], cell apoptosis was determined using Annexin V-APC/7-AAD kit (eBioscience, San Diego, CA, USA). In brief, cells were resuspended in a 1 × binding buffer with a density of 1 × 10^6^ cells/ml. Then, 5-μl Annexin V-APC and 5-μl 7-AAD were added to the cell suspension. After incubation for 15 min at room temperature in the dark, 400-μl 1× binding buffer was added. The situation of cell apoptosis was analyzed using the FACS Calibur flow cytometer (BD Biosciences, San Jose, CA, USA).

### 2.5. Luciferase reporter assay

According to previous studies [21], target sequences of 3ʹuntranslated regions (UTR) region of TULP3 of wild-type and mutant-type were cloned to pmirGLO vector (Promega) via GenePharma. Then, miR-506 mimics and reporter vector were co-transfected into 293 T cells. The interaction between miR-506 and the target gene TULP3 was determined based on the comparative fluorescent value following the introduction of the Dual-Luciferase® Reporter Assay System (Promega).

### 2.6. Subcutaneous tumor formation in nude mouse

Based on previous research [22], the lentivirus of pcDNA3.1-TULP3 (or pcDNA3.1) was bought from GenePharma. The A549 cells were injected into the abdomen of the nude mice. Until the tumor grew to 4 mm × 4 mm in size, the lentivirus at 1 × 10^7^ TU and miR-506 agomiR were injected into the tumor. Then, the nude mice treated for 17 d were sacrificed, and the tissues were removed. The tumor weight and size of the tissues were then measured. This experiment was approved by the committee of Shenzhen Bao’an Hospital of Traditional Chinese Medicine.

### 2.7. Immunohistochemistry (IHC)

As shown in the research [23], IHC was performed following standard protocol. The primary antibodies were as follows: BCL-2 (#3498, 1:100; CST, Danvers, MA,USA), Bax (#50599-2, 1:500; Proteintech, Wuhan, China), BCL-XL (#2764, 1:1200; CST), and TULP3 (#13637, 1:500; Proteintech).

### 2.8. Real-time quantitative polymerase chain reaction (RT–qPCR)

MiRNA and mRNA levels were verified by RT–qPCR based on a previous study [24]. Firstly, the RNA was collected using TRIzol (Invitrogen, Carlsbad, CA, USA). Then, complementary DNA was synthesized using M-MLV Reverse Transcriptase (Promega). Finally, a PCR reaction was performed using GoTaq qPCR Master Mix (Promega) on the ABI 7500 system (Applied Biosystem, Foster city, CA, USA). Glyceraldehyde-3-phosphate dehydrogenase was used as an internal control for mRNA detection, whereas U6 was used as reference of miRNA. Primer sequences are showed in Table 1.

**Table 1.** Primer sequences used in this study

Primer name	Sequences (5ʹ to 3ʹ)
hsa-mir-506-F	AACACGCTATTCAGGA AGGTG
Universe-R	GTGCAGGGTCCGAGGT
hsa-mir-506-RT	GTCGTATCCAGTGCAGGGTCCGAGG TATTCGCACTGGATACGACTTAAGT
hsa-U6-F	CTCGCTTCGGCAGCACA
hsa-U6-R	AACGCTTCACGAATTTGCGT
H-TULP3-F	CTGAAAACACCGTGGATACTGC
H-TULP3-R	TGGCTTGATGCAGAATTGGG
H-BCL2-F1	TCATGTGTGTGGAGAGCGTC
H-BCL2-R1	GTGCCGGTTCAGGTACTCAG
H-Bax-F	CCCGAGAGGTCTTTTTCCGAG
H-Bax-R	GCCTTGAGCACCAGTTTGC
H-Bcl-XL-F	GACTGAATCGGAGATGGAGACC
H-Bcl-XL-R	GCAGTTCAAACTCGTCGCCT
H-GAPDH-F	GAGTCAACGGATTTGGTCGT
H-GAPDH-R	GACAAGCTTCCCGTTCTCAG

### 2.9. Western blotting

We performed Western blotting according to a previous study [25]. In short, cells were lysed using RIPA reagent (Beyotime Biotechnology, Shanghai, China). The protein was quantified using the bicinchoninic acid method (Beyotime Biotechnology). The protein was run on 8% sodium dodecyl sulfate,sodium salt polyacrylamide gelelectrophoresis and then was transferred to polyvinylidene fluoride membrane (Promega). Then, the membrane was blocked by nonfat milk and incubated with primary antibody (BCL-2, #3498, 1:1000, CST; Bax, #50599-2, 1:5000, Proteintech; BCL-XL, #2764, 1:1000, CST; TULP3, #13637, 1:1000, Proteintech; cytochrome C, #ab90529, 1:1000, Abcam, Cambridge, MA, USA; and cleaved caspase-9, #ab2324, 1:1000, Abcam). The membrane was washed twice and incubated with a secondary antibody for 2 h (1:1000, Forevergen, Guangzhou, China). The membrane was washed thrice. The membrane was observed using ECL (Forevergen).

### 2.10. Statistical analysis

GraphPad Prism software (7.0; La Jolla, CA, USA) was used for the statistical analysis. P value of < 0.05 was considered significant.

## 3. Results

MiR-506 inhibits the cell cycle progression and angiogenesis of NSCLC cells, TULP3 is a potential target gene of miR-506 through the results of bioinformatics analysis. Besides, TULP3 modulate the carcinogenesis of PDAC. Therefore, we speculated that miR-506 maybe inhibite NSCLC progress by regulating TULP3. To verify our hypothesis, in vivo and in vitro experiments were performed to explore the function and potential regulatory relationship of miR-506 and TULP3 in NSCLC.

### 3.1. miR-506 inhibits viability and induces mitochondrial apoptosis of NCI-H1299 and A549 cells

**Figure 1.**
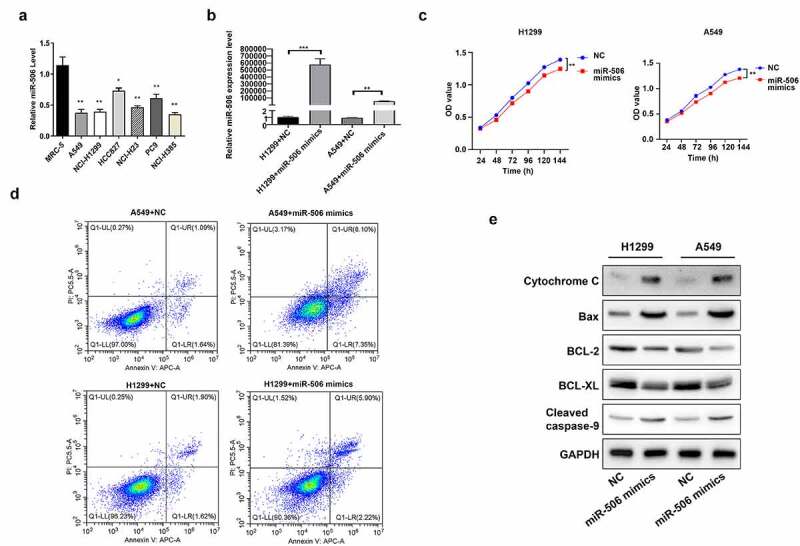
MiR-506 suppresses viability and enhances mitochondrial apoptosis of nonsmall-cell lung cancer cells. (a). miR-506 expression level in different cell lines. (b). Relative miR-506 expression level when cells were transfected with miR-506 mimics. (c). Cell viability detected using 3-(4,5-dimethylthiazol-2-yl)-5-(3-carboxymethoxyphenyl)-2-(4- sulfophenyl)-2 H-tetrazolium assay in H1299 and A549 cells. (d). Overexpression of miR-506 enhanced cell apoptosis in H1299 and A549 cells. (e). Western blotting used to determine the protein level of cytochrome C, Bax, BCL-2, BCL-XL, and cleaved caspase-9 in miR-506 mimics-transfected H1299 and A549 cells

Firstly, we confirmed the expression change of miR-506 in NSCLC cells. As shown in the RT–qPCR results, compared with MRC-5 cells, NSCLC cell lines (including A549, H1299, HCC827, NCI-H23, PC9, and NCI-H385) demonstrated lower expression of miR-506 (Figure 1a). Among NSCLC cell lines, A549 and H1299 cells showed a comparatively lower RNA level of miR-506 against others (Figure 1a). Therefore, these two cell lines were applied in the following assays. Then the miR-506 mimics was transfected into A549 and H1299 cells followed with the functions testing. By detecting the RNA level of miR-506 in cells, it was observed that the level of miR-506 was upregulated in both H1299 and A549 cells transfected with miR-506 mimics than those cells transfected with NC (Figure 1b). A time course from 24 h to 144 h was set up to evaluate the effect of miR-506 on cell viability. And the results indicated that cell viability was reduced when miR-506 mimics were induced to H1299 and A549 compared to those in cells transfected with NC (Figure 1c). Additionally, compared with the NC group, the H1299 and A549 transfected with miR-506 mimics presented a higher cell apoptosis rate than in NC group (Figure 1d). Furthermore, Western blotting was performed to determine the protein levels of apoptosis-related genes, including cytochrome C, Bax, BCL-2, BCL-XL, and cleaved caspase-9. As indicated in the results, the protein levels of BCL-2 and BCL-XL were downregulated expression, whereas cytochrome C, Bax, and cleaved caspase-9 was upregulated expression in H1299 and A549 cells transfected with miR-506 mimics compared to those cells transfected with NC (Figure 1e).

### 3.2. miR-506 conducted its functions by directly targeting TULP3 in NCI-H1299 and A549 cells

**Figure 2.**
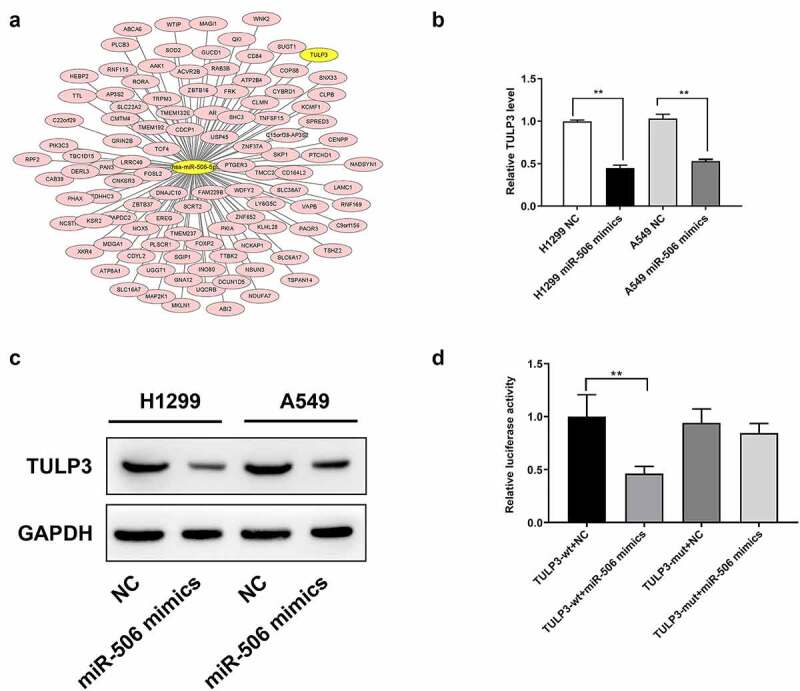
Tubby-like protein 3 is a target gene of miR-506. (a). Tubby-like protein 3 (TULP3) exhibited is a potential target gene of miR-506 in the prediction. (b). Real-time quantitative polymerase chain reaction results suggest that TULP3 expression is downregulated under the effect of miR-506 mimics. (c). Western blotting was used to detect protein level of TULP3. (d). Dual-luciferase assay proved that TULP3 is the directly target gene of miR-506

To further investigate the potential mechanism of miR-506, the downstream target genes of miR-506 were first predicted (Figure 2a). Among the targets, TULP3 showed the most binding sites than others (Figure 2a). Then we detected TULP3 expression using RT–qPCR. TULP3 expression was decreased in H1299 and A549 cells transfected with miR-506 mimics compared to those cells transfected with NC (Figure 2b). The protein level of TULP3 was also reduced in the miR-506 mimics group compared than that in NC group, which is consistent with the RT–qPCR results (Figure 2c). To validate whether the downregulation of TULP3 is due to the functions of miR-506 mimics, the luciferase reporter was designed and transfected into 293 T cells. A dual-luciferase assay was performed to determine the relative luciferase activity. For the reporter with wild-type sequences of TULP3, when the corresponding mimics increased the level of miR-506, the luciferase activity was significantly reduced (Figure 2d). However, for the reporter with mutated sequences of TULP3, the luciferase activity was not significantly changed (Figure 2d). Therefore, it proved that miR-506 can directly bind to TULP3 and downregulate its level in terms of mRNA and protein.

### 3.3. Silence of TULP3 prevents viability and aggrandizes mitochondrial apoptosis of NCI-H1299 and A549 cells

**Figure 3.**
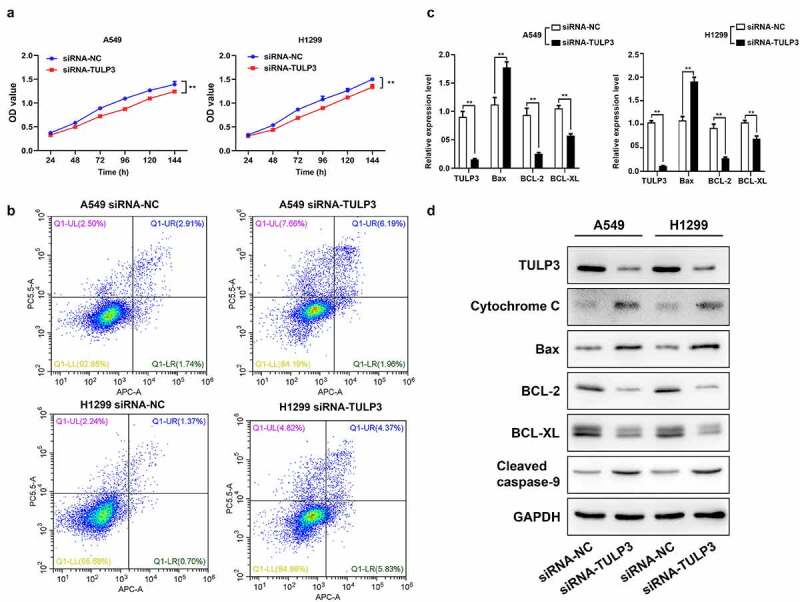
Tubby-like protein 3 silencing prevents viability and aggrandizes mitochondrial apoptosis of nonsmall-cell lung cancer cells. H1299 and A549 cells were transfected with tubby-like protein 3 (TULP3) siRNAs and siRNA-negative control (siRNA-NC), respectively. (a). Cell viability was assessed by 3-(4,5-dimethylthiazol-2-yl)-5-(3-carboxymethoxyphenyl)-2-(4- sulfophenyl)-2 H-tetrazolium assay. (b). Cell apoptosis was verified using flow cytometry. (c). Real-time quantitative polymerase chain reaction analysis of TULP3, Bax, BCL-2, and BCL-XL. (d). Western blotting of TULP3, cytochrome C, Bax, BCL-2, BCL-XL, and cleaved caspase-9 expressions

Based on the targeted downregulation of miR-506 to TULP3 expression, it was further verified whether silencing TULP3 can also restrain viability and accelerate mitochondrial apoptosis like that of miR-506 overexpression. Firstly, MTS data signified that cell viability was observably attenuated in TULP3-silenced H1299 and A549 cells relative to that in the siRNA-NC group (Figure 3a). Secondly, the flow cytometry results denoted that TULP3 silencing could cause a prominent enhancement of apoptosis in H1299 and A549 cells transfected with siRNA-TULP3 compared to those in siRNA-NC group (Figure 3b). Thirdly, RT–qPCR and Western blotting results showed that knockdown of TULP3 could dramatically downregulate TULP3, BCL-2, and BCL-XL and upregulate cytochrome C, Bax, and cleaved caspase-9 relative to that in the siRNA-NC group (Figure 3c and d).

### 3.4. MiR-506 suppresses NSCLC progression by regulating TULP3

**Figure 4.**
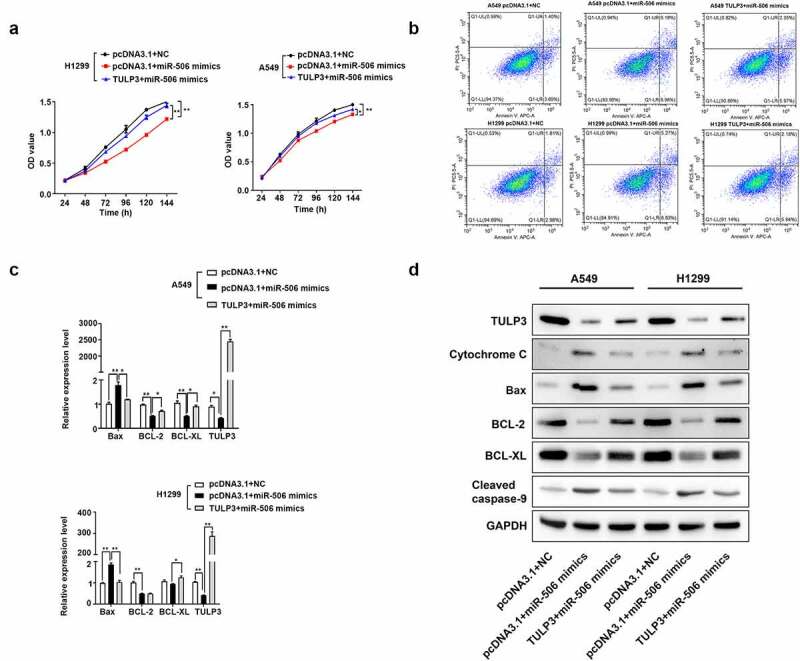
MiR-506 depresses nonsmall-cell lung cancer progression by tubby-like protein 3. (a). 3-(4,5-dimethylthiazol-2-yl)-5-(3-carboxymethoxyphenyl)-2-(4- sulfophenyl)-2 H-tetrazolium assay was used to determine cell viability for the cells H1299 and A549 under the treatment of miR-506 mimics and tubby-like protein 3 (TULP3) overexpression vector. (b). Flow cytometry to determine the cell apoptosis under the treatments of overexpression of miR-506 and TULP3. (c). Real-time quantitative polymerase chain reaction was used to determine the mRNA level of the cell apoptosis-related genes under the treatments of overexpression of miR-506 and TULP3. (d). Western blotting was used to determine the protein levels of mitochondrial apoptosis-related proteins under the treatments of overexpression of miR-506 and TULP3

The rescue experiment was adopted to further determine the regulatory relationship between miR-506 and TULP3 in NSCLC progression. MTS assay was firstly conducted to determine cell viability. As presented in the results, when miR-506 mimics and pcDNA3.1 were transfected into H1299 and A549 cells, cell viability decreased significantly compared with the pcDNA3.1+ NC group (Figure 4a). However, for cells transfected with the TULP3 overexpression vector and miR-506 mimics, cell viability was increased compared to those cells transfected with miR-506 mimics and pcDNA3.1 (Figure 4a). Additionally, cells transfected with pcDNA3.1 and miR-506 mimics showed an obviously increased percentage of apoptotic cells compared with the pcDNA3.1+ NC group (Figure 4b). Compared with the cells transfected with empty pcDNA3.1 vector and miR-506 mimics, cells transfected with TULP3 overexpression vector and miR-506 mimics showed lower percentage of apoptotic cells (Figure 4b). Furthermore, cells transfected with empty vector and miR-506 mimics presented higher expression levels of cytochrome C, Bax, and cleaved caspase-9 but lower expression levels of TULP3, BCL-2, and BCL-XL compared with cells transfected with pcDNA3.1+ NC (Figure 4c and d). When the cells were transfected with TULP3 overexpression vector and miR-506 mimics, the expression levels of cytochrome C, Bax, and cleaved caspase-9 were slightly reduced, but TULP3, BCL-2, and BCL-XL expression were increased compared with cells transfected with empty vector and miR-506 mimics (Figure 4c and d). Therefore, the evidence above proved that TULP3 could attenuate the effect of miR-506 mimics on cell viability and mitochondrial apoptosis of H1299 and A549 cells.

### 3.5. MiR-506 restrains growth and induces mitochondrial apoptosis in the mice xenograft model of NSCLC by regulating TULP3

**Figure 5.**
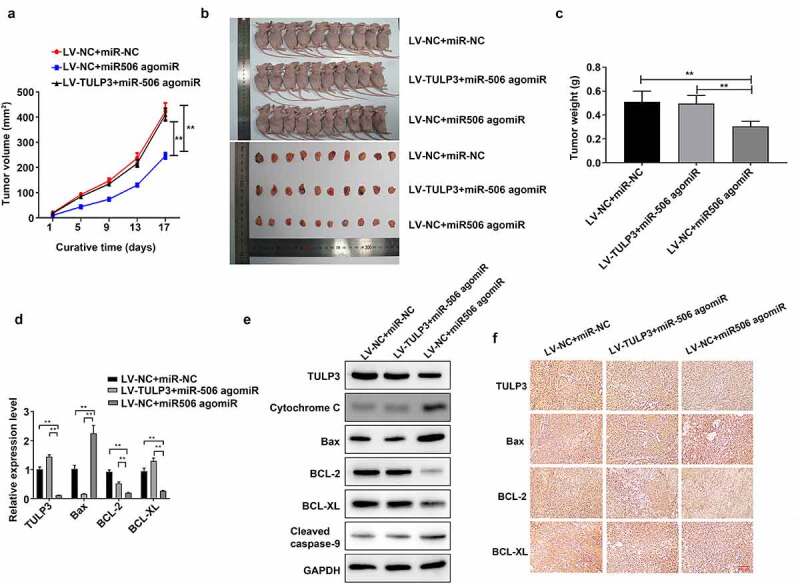
MiR-506 restrains growth and induces mitochondrial apoptosis in the mice xenograft model of nonsmall-cell lung cancer. (a). Tumor volume under the effect of tubby-like protein 3 (TULP3) and miR-506 overexpression. (b). Photographs of the nude mice and tumors under the effect of TULP3 and miR-506 overexpression. (c). The miR-506 agomiR reduced the weight of the tumor, but the overexpression of TULP3 reversed this effect. (d). Real-time quantitative polymerase chain reaction was used to determine the mRNA levels of TULP3 and apoptosis-related genes under the treatments of overexpression of miR-506 and TULP3. (e). Western blotting was used to determine the levels of TULP3, mitochondrial apoptosis-related proteins under the treatments of overexpression of miR-506 and TULP3. (f). Immunohistochemical staining was used to determine the protein level of the cell apoptosis-related protein under the treatments of overexpression of miR-506 and TULP3

Subsequently, the role of miR-506 in the mice xenograft model of NSCLC was further validated. The tumor volume was measured on days 1, 5, 9, 13, and 17. As indicated in the results, LV-NC + miR-506 agomiR significantly reduced the tumor volume compared to LV-NC + miR-NC group, whereas LV-TULP3 + miR-506 agomiR reversed the effect of LV-NC + miR-506 agomiR on tumor volume (Figure 5a). The mice were sacrificed on day 17, the tumor size was smaller in LV-NC + miR-506 agomiR group than those in LV-NC + miR-NC group, but the effect of LV-NC + miR-506 agomiR on tumor size was reversed by LV-TULP3 + miR-506 agomiR (Figure 5b). Similarly, the tumor weight was decreased in LV-NC + miR-506 agomiR group than those in LV-NC + miR-NC group, while LV-TULP3 + miR-506 attenuated the effect of LV-NC + miR-506 agomiR on tumor weight (Figure 5c). Additionally, the results showed that miR-506 agomiR notably raised the level of Bax but reduced TULP3, BCL-2, and BCL-XL expressions compared to those in LV-NC + miR-NC group, whereas the expression changes of these four indicators mediated by miR-506 agomiR could be dramatically attenuated by TULP3 overexpression in the mice tumors (Figure 5d and e). Furthermore, TULP3 overexpression could prominently weaken upregulation of cytochrome C and cleaved caspase-9 mediated by miR-506 agomiR in the mice tumors (Figure 5e). Besides, the tumors were evaluated using immunohistochemical staining. The positive signal in the section showed the level of specific genes. It demonstrated results that were consistent with Western blotting: miR-506 agomiR raised protein levels of Bax but reduced protein level of TULP3, BCL-2, and BCL-XL compared to those in LV-NC + miR-NC group, whereas TULP3 overexpression vector reversed the effect of miR-506 agomiR on the protein level of TULP3, BCL-2, and BCL-XL (Figure 5f).

## 4. Discussion

In this study, in vitro cell assays and in vivo tumor generation assays were conducted to validate the functions of miR-506 and its downstream target gene TULP3. It was found that miR-506 acts as a tumor suppressor that can inhibit cell viability and induce cell mitochondrial apoptosis of NSCLC by regulating TULP3.

It has been reported that miR-506 can regulate cell cycle progression and angiogenesis of NSCLC in in vitro assays [12]. The underlying mechanism targets cyclin dependent kinase (CDK) 1, 4, and 6 genes, and the effect blocks the G1 and G2 cell cycle, thereby suppressing cell proliferation. Alternatively, miR-506 can cause cytotoxic activity by activating caspase 3/7. Therefore, it is consistent with the results of this study that upregulated expression of miR-506 induces cell mitochondrial apoptosis and suppresses cell viability. Apart from NSCLC, there are some studies suggesting similar functions of miR-506 in other cancers. For osteosarcoma, miR-506 was reduced in patients’ specimens. Meanwhile, the gene expression of Snail was increased compared with normal bone tissue. Under this condition, cell invasiveness was observed in osteosarcoma. With the overexpression of miR-506, the expression level of Snail was significantly reduced, and cell invasiveness was suppressed [26]. Another study on hepatocellular carcinoma showed that miR-506 contributes in inhibiting cell proliferation by inducing G1/S cell cycle arrest and cell apoptosis. It was proved that the downstream target of miR-506 is rho associated coiled-coil containing protein kinase 1 (ROCK1). The upregulated ROCK1 can reverse the suppressive effects of miR-506 in hepatocellular carcinoma [27]. In ovarian carcinoma, miR-506 acted as a suppressor of tumorigenesis. It inhibited CDK4/6-forkhead box M1 signaling and thereby inhibited cell proliferation and promoted senescence [28,29]. It was also found that miR-506 was downregulated and expressed in clear-cell renal cell carcinoma. Suppression of miR-506 was associated with an advanced clinical stage and poor prognosis [30]. Therefore, according to evidences, miR-506 might be a tumor suppressor for broad types of carcinoma, but the regulatory mechanism might vary across different carcinomas.

8TULP3 was validated as the downstream target of miR-506 in our study. The expression level of TULP3 is positively correlated with cell viability and suppression of cell mitochondrial apoptosis. Overexpression of TULP3 can reverse the suppressive effect of miR-506. To the best of our knowledge, there is currently no study suggesting a correlation between TULP3 and NSCLC. Studies so far have shown that TULP3 is a member of a small gene family consisting of TUB bipartite transcription factor (Tub) and TUB like protein (Tulp)s1-3 correlated with the maintenance and function of neural cells [31]. TULP3 is reported to be a member of the Tub gene family that contributes to embryonic development. The TULP3 -/- embryos die during gestation [32]. The evidence suggests that TULP3 is essential in regulating the processes of cell growth and development. A previous study also indicated that TULP3 plays a role as a regulator of carcinogenesis in PDAC, and higher expression of TULP3 is correlated with poor prognosis [15]. Another study also showed that high expression of TULP3 was associated with lymphatic and vascular invasion in colon and rectal adenocarcinomas [16]. From these results, it was proved that the expression of TULP3 might be positively correlated with the proliferation of NSCLC cells, which is consistent with the findings of our study. Besides, it was discovered that the knockdown of TULP3 could reduce BCL-2 and BCL-XL expression and increase Bax, cytochrome C, cleaved caspase-9 expression in A549 and H1299 cells, and the interference of TULP3 could markedly enhance the mitochondrial apoptosis of A549 and H1299 cells. Therefore, TULP3 can be a potential direct or indirect target of miR-506 that regulates the processes of NSCLC.

## 5. Conclusions

Our study was firstly provided evidence of the potential regulatory cascade of miR-506 and TULP3 in the processes of NSCLC. In conclusion, miR-506 inhibits the development of NSCLC by regulating TULP3. However, the current research has certain limitations such as the signaling pathway regulated by TULP3 is need further validation, the expression of miR-506 and TULP3 in clinical tissues samples is also need further confirm. Therefore, more experiments are needed to perform to confirm the role and potential mechanism of miR-506 and TULP3 in NSCLC.

## Data Availability

The data that support the findings of this study are available from the corresponding author upon reasonable request.
